# The Imperfect Development of the Uterus

**Published:** 1904-04

**Authors:** R. R. Kime

**Affiliations:** Atlanta, Ga., Gynecologist to Tabernacle Infirmary; Ex-President Tri-State Medical Society Alabama, Georgia and Tennessee; Georgia State Sociological Society, and Atlanta Society of Medicine


ATLANTA

### Journal-Record of Medicine.

Successor to Atlanta Medical and Surgical Journal, Established 1855, and Southern Medical Record, Established 1870.

VOL. VI. APRIL, 1904. No. 1.

BERNARD WOLFF, M.D., EDITOR, Nos. 319-20 Prudential.

Published Monthly.

M. B. HUTCHINS, M.D., BUSINESS MANAGER, 1007-1008 Century Bldg.

### ORIGINAL COMMUNICATIONS.

### THE IMPERFECT DEVELOPMENT OF THE UTERUS.*

BY R. R. KIME, M.D., ATLANTA, GA.,

Gynecologist to Tabernacle Infirmary; Ex-President Tri-State Medical Society Alabama, Georgia and Tennessee; Georgia State Sociological Society, and Atlanta Society of Medicine.

Taken in its broadest sense this subject would take more time and study than we have at our disposal.

We will not enter into the histogenesis of the development of the uterus nor its various malformations, but try to present a few practical thoughts on the management of those cases where Mullers ducts have completely coalesced, forming the normal outlines of uterus and adnexa but not reaching full development. Such conditions include infantile uteri, small uteri with or without anteflexion or retroflexion, with irregular, imperfect menstrual periods, and at times amenorrhœa. In this day and age in city life such conditions are becoming more common, while country life with pure air

*Read before the Atlanta Society of Medicine, March 3, 1904.

and normal living favors physical development and prevention of these conditions.

All physicians recognize the necessity of proper physical development of girls from the age of ten to sixteen.

It is also recognized that the generative organs develop earlier in warm than cold climates, thus reaching puberty at an earlier age. It is evident to observers that girls living in cities should get more fresh air, physical exercise, and in many instances better diet and more appropriate clothing. Many a weakly child is crowded into school by the time she is six, or before, and pushed from year to year as rapidly as possible, often adding music and other accomplishments extra. In many instances parents indulge their children in night entertainments and social dissipations that only add fuel to the fire that consumes their physical vitality.

The school board and teachers of this city contribute their quota to this process of physical degeneration by long daily sessions and overtaxing. Something is radically wrong in a school system that takes children and young girls from home at 8 to 8:15 a.m., with a hurried breakfast often illy prepared, and many times as imperfectly digested, and keeps them at the schoolroom from 8:30 to from 1:30 to 2 p.m., requiring them to do without dinner or food until 2:30 or 3:00 p.m. If they take a lunch it is often of a very undigestible character. Even with a lunch it makes breakfast at 7:30 or 8 a.m., lunch at 11:30 or 12 m., dinner or lunch at 2:30 to 3 p.m., and another meal at 6 or 7 p.m.

It would take a stomach like an ostrich to keep up such a course for eight to ten years without injury.

Can a child do good mental work under such conditions and properly develop physically? Could a stout, hearty, well-developed man do good mental work for so many hours under similar conditions for ten or twelve years without injury?

It is a well-recognized fact that girls overtaxed physically or mentally and on insufficient diet at certain ages fail to develop properly. The pelvic organs fail to receive proper nourishment for normal physiological growth and are controlled by perverted nerve functions, thus developing disease or weak, imperfect structures. After the school age the customs and demands of society add fuel to the fire.

Thus we have so many puny, weakly, sickly, finicky hysterical women unfit for maternity and the duties of home and motherhood.

It is time we, as a people, in cities especially, were looking more after the physical development of the race. Not the development of professional pugilists, professional ball-players, but the normal physiological development of the masses, including the girls and young women.

This we can not do so long as the men dissipate so freely and the women are raised as hothouse plants. To be more explicit and come more directly to the subject, we find in many of these cases of the imperfectly developed a small uterus at times infantile in character. It is frequently anteflexed or retroflexed, cervix longer and larger in proportion to the fundus than in normal conditions. In the true infantile uterus the cervix may be twice the size and length of the body.

With a small uterus it is not unreasonable to suppose that the adnexa—tubes and ovaries—participate in the same deficient development in some cases. We have here a specimen of an infantile uterus taken from a girl of thirteen that died of anæmia and chronic interstitial nephritis. Had been in bad health for one or two years before death. Nephritis result of scarlet fever two years previous; menstruated once about one year before death—only menstruation; had some four or five hemorrhages from gums, stomach and bowels about one month apart; had chronic malaria several years previous; never a very robust child.

We find but little written in our text-books upon the treatment of these cases; so little, one would conclude nothing could be done.

From practical clinical experience I am inclined to believe many of these cases can be greatly benefited, if not permanently relieved. To illustrate I will give a brief report of a few cases:

Mrs. F., aged 20, married two and a half years; no flow during last year; always very irregular and very scant; in poor health, weighing less than 100 pounds; uterus very small, retroflexed, tender; ovaries apparently small; dilated cervix; right ovary slightly prolapsed; curetted uterus, followed with gauze strip then used intra-uterine stem for about four months.

Gave tonics, good nourishment; patient became pregnant and is now the mother of three children and weighs 125 to 130 pounds, and in good health.

Case 2. Miss Z., aged 20, from out of city; referred to me by an oculist, being under his treatment for retinal disease, probably hemorrhage and error of refraction. Had to quit school on account of eyes and general health. Could not find any disease of kidneys or other organs to account for condition except a small anteverted, tender, inflamed uterus; menstrual periods irregular, scant, at times absent with tendency to hemorrhages elsewhere.

Dilated, curretted and followed with gauze strip; later with intra-uterine stem for four months. Had to adjust a hard-rubber Smith-Hodge retroversion pessary to keep stem in position.

Patient improved rapidly and gained fifteen or twenty pounds in flesh and returned to school.

Case 3. Miss R., aged 26; in poor health; very irregular, scant flows; none in six months. Uterus small, markedly anteflexed and somewhat prolapsed; left tube and ovary inflamed and tender.

Dilated, curetted, making transverse incision in vaginal wall between cervix and body, straightened uterus well, then put incision on the stretch with tenaculæ and sewed it up with longitudinal axis of vagina, just opposite to the direction incision was made. Followed with usual treatment; patient very much improved in general health and local condition.

Case 4. Miss N., aged 20; uterus small, retroverted; periods irregular, scant and painful; general health bad.

Dilated, curetted, followed by usual treatment and uterine support. Patient in good health and gained fifteen or twenty pounds in weight.

Do not understand that I advise pelvic examination in girls and young women as a routine or as a common practice—far from it. Such examinations and treatment should only be resorted to when all other means fail.

Simple dilatation and curettage will not relieve most of these cases, as they need other treatment. The presence of the intrauterine stem stimulates uterine action, and by its presence also increases blood supply to the organ, thus favoring increased development. Fortunate are these cases if they are married and pregnancy supervenes. In many instances nature can do better work than we by the physiological development that takes place during pregnancy, while displacements can be corrected by appropriate care after delivery. In some cases the flexion is very marked, the cervix and fundus almost pressing against each other; in such the transverse incision and stitching is of marked benefit.

The best age to correct these conditions when local treatment is necessary, is from eighteen to twenty-five; the older the less likely to get good results.

My usual method of treating imperfect development of uterus is as follows:

Prepare patient as for any vaginal operation with due regard for aseptic work. Under anesthesia dilate cervix and curette womb. If marked flexion exists make transverse incision in vaginal wall and straighten womb, approximate incised tissues in long axis of vagina by use of tenaculæ; stitch with interrupted catgut sutures. Dress uterine cavity with strip of aseptic gauze, the end moistened with pure camphorated phenol gauze, removed on second or third day; hot boric acid douche (two gallons) used once or twice a day, and patient kept in bed seven to ten days.

After patient is over immediate effect of operation, antiseptic strips of gauze are used, changed every second day until uterus is accustomed to the presence of a foreign body, then a hard-rubber intra-uterine stem is adjusted. If it will not remain of itself then a proper hard-rubber support is adjusted. The stem and support are removed and cleansed every two to four weeks and worn until the desired results are attained. During local treatment general tonics are given—iron if needed, good nourishing diet, fresh air and proper exercise.

All functions of the various organs of the body are regulated as near as possible, giving laxatives if needed.

In the treatment of these cases the suggestion of Goodell is applicable: "It is well to remember that a woman has other organs besides the pelvic organs."

